# ﻿Assigning *Xiuguozhangia* (genus *incertae sedis*) to Pseudoberkleasmiaceae (Dothideomycetes, Pleosporomycetidae, Pleosporales) and introducing *X.broussonetiae* as a novel species

**DOI:** 10.3897/mycokeys.111.136202

**Published:** 2024-12-20

**Authors:** Deecksha Gomdola, Rajesh Jeewon, Eleni Gentekaki, Ruvishika S. Jayawardena, Kevin D. Hyde, Fatimah Alotibi, Yong Wang

**Affiliations:** 1 Department of Plant Pathology, College of Agriculture, Guizhou University, Guiyang, Guizhou 550025, China; 2 School of Science, Mae Fah Luang University, Chiang Rai 57100, Thailand; 3 Center of Excellence in Fungal Research, Mae Fah Luang University, Chiang Rai 57100, Thailand; 4 Department of Health Sciences, Faculty of Medicine and Health Sciences, University of Mauritius, Réduit 80837, Mauritius; 5 Department of Zoology, College of Science, King Saud University, P.O. Box 22452, Riyadh 11495, Saudi Arabia; 6 Department of Veterinary Medicine, University of Nicosia School of Veterinary Medicine, Cyprus 24005, Cyprus; 7 Department of Botany and Microbiology, College of Science, King Saud University, P.O. Box 22452, Riyadh 11495, Saudi Arabia

**Keywords:** 1 new taxon, hyphomycetes, morphology, phylogeny, sequence data, taxonomy

## Abstract

*Xiuguozhangia* species are dematiaceous hyphomycetes that are characterised by acropleurogenous, dictyoseptate, campanulate or cheiroid, and brown to dark brown conidia that are composed of several layers of cells radiating from a protuberant basal cell, and mostly seen with appendages arising from the apical cells. The genus was introduced based on morphology to accommodate five of the six *Piricaudiopsis* species that exhibited holoblastic conidial ontogeny. *Xiuguozhangia* was referred to as Ascomycota genus *incertae sedis* as it was challenging to resolve its taxonomic placement based solely on the available morphological data (no DNA sequence data was previously available). In this study, we provide DNA sequence data for LSU, ITS, SSU, *TEF1*, and *RPB2* for our isolates, collected from *Broussonetiapapyrifera* (Moraceae) in northern Thailand. Based on morphology, we classify our isolates as *Xiuguozhangia*. Since they form a sister lineage to *Pseudoberkleasmium*, we place *Xiuguozhangia* in Pseudoberkleasmiaceae (Pleosporales). Within *Xiuguozhangia*, we establish these two isolates as a new taxon, *Xiuguozhangiabroussonetiae*, in view of the presence of new conidiogenous cells developing from subtending cells. *Xiuguozhangiabroussonetiae* differs from the extant species in the genus as it has longer conidiophores that are sometimes branched, comprising numerous septa, and its appendages are mostly untapered (sometimes tapering) towards the tips, a feature not observed in other *Xiuguozhangia* species. This is the first study to provide DNA sequence data and phylogenetic relationships for *Xiuguozhangia*. Furthermore, we analysed selected DNA sequence data and provided an updated phylogenetic tree incorporating all families (with representative genera) of Pleosporales.

## ﻿Introduction

*Xiuguozhangia* was introduced by Zhang et al. (2014) based on morphological characteristics and typified with *X.rosae*. The genus is characterised by effuse, hairy, and brown to dark brown colonies on natural substrates. Conidiophores are erect, straight or flexuous, multiseptate, and brown to dark brown at the base, becoming pale brown towards the apex. Conidiogenous cells are monoblastic, terminal or integrated, lageniform, and pale brown, displaying zero to several percurrent proliferations. In addition, their conidia are acropleurogenous, dictyoseptate, campanulate, cheiroid and brown, comprising multiple layers of cells, either with or without appendages (Zhang et al. 2014; Liu et al. 2024b).

Initially, five *Xiuguozhangia* species were established, all of which are combinations of *Piricaudiopsisviz*., *Xiuguozhangiaappendiculata*, *X.indica*, *X.punicae*, *X.rhaphidophorae* and *X.rosae*. *Piricaudiopsis* was established by Mena and Mercado (1987) based on morphology and typified with *P.elegans*. The new combinations of *Xiuguozhangia* were proposed based on their holoblastic conidial ontogeny; *Piricaudiopsis* was reported to have enteroblastic conidial ontogeny (Zhang et al. 2014; Dubey and Jash 2024). However, this morphological difference based on conidial ontogeny is subtle, necessitating careful consideration before applying this trait in generic delineation. Given the limited taxon sampling and analysis across both genera, coupled with the lack of DNA sequence data, it is still uncertain whether *Xiuguozhangia* is actually distinct from *Piricaudiopsis*, or if they could represent the same genus. Both genera were referred to as Ascomycota genera *incertae sedis* in the latest outlines by Wijayawardene et al. (2022a) and Hyde et al. (2024c) as it was difficult to establish their precise taxonomic placement without the availability of DNA sequence data. Recently, based on morphological description, a new species was added to *Xiuguozhangiaviz*., *X.macrospora*, collected from dead bamboo stems in India (Dubey and Jash 2024). To date, all extant species of *Xiuguozhangia* have been described as saprobes and collected from China and India (Zhang et al. 2014; Dubey and Jash 2024), while no studies have reported its occurrence in Thailand.

The highlights of this study are as follows: (i) We provide DNA sequence data for multiple gene regions (LSU, ITS, SSU, *TEF1*, and *RPB2*) for our isolates, collected from *Broussonetiapapyrifera* (Moraceae) in northern Thailand; (ii) Based on morphology, we classify our isolates as *Xiuguozhangia*. Since they form a sister lineage to *Pseudoberkleasmium*, we place *Xiuguozhangia* in Pseudoberkleasmiaceae (Pleosporales); (iii) Within *Xiuguozhangia*, these two isolates could potentially represent a new taxon, *Xiuguozhangiabroussonetiae* in view of the presence of new conidiogenous cells developing from subtending cells. This is the first study to provide DNA sequence data and phylogenetic relationships for *Xiuguozhangia*. In addition, we analysed selected sequence data from GenBank and provided an updated phylogenetic tree incorporating all families (with representative genera) of Pleosporales.

## ﻿Materials and methods

### ﻿Sample collection and examination, material deposition, and species delimitation methods

Decaying stems of *Broussonetiapapyrifera* (Moraceae) colonised by hyphomycetes were collected from deciduous forest in the Mae Fah Luang University Botanical Garden, Chiang Rai, Thailand during the dry, hot season; collection information was noted (Rathnayaka et al. 2024) and brought to the laboratory in paper boxes for further characterisation (Senanayake et al. 2020). Morphological characteristics were observed using a Motic SMZ 168 Series stereo-microscope. Several conidia were picked from the colonies on the substrate using a sterile needle and placed directly on fresh potato dextrose agar plates (PDA, 39 g/L) containing antibiotics (Amoxicillin, MacroPhar). Conidium germination was observed within 48 hours, and pure cultures were incubated for four weeks at 25 °C under dark conditions. Digital images of micro-morphological characters were captured using a Cannon 750D camera (Canon, Tokyo, Japan) attached to a Nikon ECLIPSE E600 compound microscope (Nikon, Tokyo, Japan) based on the bright-field microscopy technique. Photo plates were assembled using Adobe Photoshop CS6 version 2020 (Adobe Systems, USA), and measurements were made using Tarosoft® Image Frame Work (version 0.97).

The holotype specimen and ex-type living culture have been deposited in the Mae Fah Luang University Herbarium (MFLU) and Mae Fah Luang University Culture Collection (MFLUCC), respectively. Faces of Fungi and Index Fungorum numbers are given for the new species (Jayasiri et al. 2015; Index Fungorum 2024). The description and illustration of the new species have also been updated in the GMS microfungi database (https://gmsmicrofungi.org/) (Chaiwan et al. 2021).

The new species is established based on the morphological species concept and complemented with the phylogenetic species concept (Maharachchikumbura et al. 2021; Pem et al. 2021). Features of *Xiuguozhangia* species are compiled, and a comparison is done to showcase the major differences across existing taxa.

### ﻿DNA extraction, PCR amplification, and sequencing

Total genomic DNA was extracted from axenic cultures grown on PDA for 28 days using the BIOMIGA Fungus Genomic DNA Extraction Kit (BIOMIGA, San Diego, CA, USA), following the manufacturer’s instructions. The internal transcribed spacer (ITS), large subunit (LSU), and small subunit (SSU), and the genes for RNA polymerase 2 (RPB2) and translation elongation factor 1α (TEF1) were amplified using the following primers: ITS1/ITS4, LR0R/LR5, NS1/NS4 for ITS, LSU, and SSU, respectively (White et al. 1990); fRpb2-5F/7CR for *RPB2* (Liu et al. 1999); and 728F/2218R for *TEF1* (O’Donnell et al. 1998; Carbone and Kohn 1999).

The polymerase chain reaction (PCR) mixture had a final volume of 20 µL, comprising 10 µL of PCR master mix, 1 µL of the forward and reverse primers each (10 µM stock concentration), 7 µL of double-distilled water, and 1 µL of the template DNA. The PCR conditions were as follows: initial denaturation at 95 °C for 3 min; denaturation at 95 °C for 45 s; annealing at 55 °C for 50 s (ITS), 52 °C for 50 s (LSU and SSU), 58 °C for 1 min 30 s (*RPB2* and *TEF1*); extension at 72 °C for 2 min; and final extension at 72 °C for 10 min (number of cycles = 40). Purification and bidirectional sequencing of PCR amplicons were carried out at Sangon Biotech (Shanghai) Co., Ltd., China.

### ﻿Phylogenetic analyses

The raw reads were checked using DNA Baser Assembler, and ambiguous bases from the 5’ and 3’ ends were trimmed manually. Consensus sequences were generated using SeqMan (DNAStar, Madison, Wisconsin, USA). The sequences have been deposited in the NCBI GenBank database, and accession numbers for all strains are provided (Table 1). Newly obtained sequences were subjected to blast searches in NCBI, and sequences of ITS, LSU, SSU, *RPB2*, and *TEF1* from other species were retrieved from GenBank (Table 1). Two different datasets were analysed in this study. The first dataset (Dataset 1) evaluated familial relationships and was based on a larger taxon sampling, incorporating representative genera with DNA sequence data from 92 families of Pleosporales. Diademaceae and Lizoniaceae, which also belong to Pleosporales, lack molecular data. Another dataset (Dataset 2) evaluated the phylogenetic relationships within and between *Xiuguozhangia* (Pseudoberkleasmiaceae) and its phylogenetically closely related genera, *Pseudoberkleasmium* (Pseudoberkleasmiaceae) and *Hermatomyces* (Hermatomycetaceae). Dataset 2 was based on multiple strains for these genera.

**Table 1.** Names, isolate numbers, and the corresponding GenBank accession numbers of taxa used in the phylogenetic analyses. Type, ex-type, and reference strains are denoted with ^T^. The new isolates are in bold font.

Species	Isolate/strain number	LSU	ITS	SSU	*TEF1*	*RPB2*
*Acrocalymmaaquatica*	MFLUCC 11-0208 ^T^	JX276952	JX276951	JX276953	–	–
*Acrocalymmapterocarpi*	MFLUCC 17 0926 ^T^	MK347949	MK347732	MK347840	MK360040	–
*Ageratinicolakunmingensis*	KUMCC 21-0217 ^T^	NG_243113	NR_191196	NG_242816	–	–
*Aigialusgrandis*	BCC 20000 ^T^	GU479775	–	GU479739	GU479839	–
*Alternariaatrobrunnea*	FMR 16868 ^T^	–	LR537033	–	LR537051	LR537044
*Alternariaalternata*	AFTOL-ID 1610 ^T^	DQ678082	KF465761	KC584507	KC584634	KC584375
*Amniculicolaaquatica*	MFLUCC 16-1123 ^T^	MK106096	–	MK106108	MK109800	–
*Amorocoelophomacassiae*	MFLUCC 17-2283	MK347956	MK347739	NG_065775	MK360041	MK434894
*Anastomitrabeculiadidymospora*	MFLU 20-0694 ^T^	MW412978	NR_172008	NG_073568	MW411338	–
*Anastomitrabeculiadidymospora*	MFLU 11-0236	ON077069	ON077080	ON077074	ON075063	ON075067
*Angustimassarinalonicerae*	MFLUCC 15-0087	KY496724	KY496759	–	–	–
*Anteagloniumgordoniae*	MFLUCC 17-2431 ^T^	MK347977	MK347761	MK347867	MK360042	MK434881
*Anteagloniumgordoniae*	CD7	–	OK335788	–	–	–
*Anteagloniumlatirostrum*	GKM1119 ^T^	GQ221874	–	–	GQ221937	–
*Anteagloniumlatirostrum*	GKML100Nb	GQ221876	–	–	GQ221938	–
*Aquadictyosporaclematidis*	MFLUCC 17-2080 ^T^	MT214545	MT310592	MT226664	MT394727	MT394679
*Aquastromamagniostiolata*	HHUF 30122 ^T^	AB807510	LC014540	AB797220	AB808486	–
*Aquasubmersajaponica*	HHUF 30469 ^T^	NG_057138	NR_154739	NG_062426	LC194384	LC194421
*Aquasubmersajaponica*	MFLUCC 17-2121	OP377971	OP377885	OP378047	OP473059	OP473118
*Aquasubmersajaponica*	MFLUCC 15-0622	OP377958	OP377872	OP378036	OP473051	OP473112
*Aquasubmersamircensis*	MFLUCC 11-0401 ^T^	NG_042699	JX276954	NG_061141	–	–
*Aquihelicascussongkhlaensis*	MFLUCC 18-1154 ^T^	MN913692	MT627680	–	MT954380	–
*Aquihelicascussongkhlaensis*	MFLUCC 18-1273	MN913724	MT627696	MT864319	MT954369	MT878464
*Aquimassariosphaeriakunmingensis*	KUMCC 18-1019 ^T^	MT627661	–	MT864312	MT954409	MT878454
*Ascocylindricamarina*	MD6011 ^T^	KT252905	–	KT252907	–	–
*Ascocylindricamarina*	MF416	MK007123	–	MK007124	–	–
*Astragalicolavasilyevae*	MFLUCC 17-0832 ^T^	MG828986	NR_157504	MG829098	MG829193	MG829248
*Astrosphaeriellafusispora*	MFLUCC 10-0555	KT955462	–	KT955443	KT955425	KT955413
*Atrocalyxglutinosus*	DAOM: 252609 ^T^	OQ400928	OQ400918	–	OQ413076	OQ413081
*Atrocalyxglutinosus*	CHEM 2721	OQ400930	OQ400920	–	–	OQ413084
*Bahusandhikaindica*	GUFCC 18001	KF460274	KF460273	–	–	–
*Bambusicolabambusae*	MFLUCC 11-0614 ^T^	JX442035	JX442031	JX442039	–	KP761718
*Berkleasmiumaquaticum* (Tubeufiales)	MFLUCC 17-0049 ^T^	KY790432	KY790444	–	KY792608	MF535268
*Berkleasmiumaquaticum* (Tubeufiales)	MFLUCC 17-0039	KY790431	KY790443	–	KY792607	MF535267
*Berkleasmiumlongisporum* (Tubeufiales)	MFLUCC 17-1999 ^T^	MH558825	MH558698	–	MH550889	MH551012
*Berkleasmiumlongisporum* (Tubeufiales)	MFLUCC 17-2002	MH558826	MH558699	–	MH550890	MH551013
*Bertiellafici*	MFLU 19-2713 ^T^	MW063223	–	MW079351	MW183786	–
*Bertiellafici*	NCYU 19-0073	MW063224	–	MW079352	MW183787	–
*Bipolarisadikaramae*	HSF070 ^T^	–	MN535176	–	MT548605	–
*Boeremialinicola*	CBS 116.76 ^T^	GU237938	GU237754	–	KY484705	KT389574
*Boeremialinicola*	CBS 248.38	KT389703	KT389486	–	–	KT389575
*Biatriosporaborsei*	NFCCI-4245 ^T^	MK358813	MK358818	MK358811	MK330938	–
*Biatriosporamarina*	CY 1228	GQ925848	–	GQ925835	GU479848	GU479823
*Brevicollumhyalosporum*	MAFF 243400 ^T^	LC271239	LC271242	LC271236	LC271245	LC271249
*Brevicollumhyalosporum*	MFLUCC 17-0071	MG602200	MG602204	MG602202	MG739516	–
*Brevicollumversicolor*	HHUF 30591 ^T^	NG_058716	NR_156335	NG_065124	LC271246	LC271250
*Brunneoclavisporacamporesii*	MFLUCC 11-0001 ^T^	MN809328	MN809329	–	–	–
*Brunneofusisporaclematidis*	MFLUCC 17-2070 ^T^	MT214570	MT310615	MT226685	MT394629	MT394692
*Camarosporiumquaternatum*	CPC 31081 ^T^	NG_064442	NR_159756	KY929123	KY929201	–
*Camarosporomycesflavigenus*	CBS 314.80 ^T^	GU238076	MH861266	NG_061093	–	–
*Camarosporidiellacaraganicola*	MFLUCCC 14-0605 ^T^	KP711381	KP711380	KP711382	–	–
*Camarosporidiellamelnikii*	MFLUCC 17-0684 ^T^	MF434250	MF434162	MF434338	MF434425	–
*Capulatisporasagittiformis*	HHUF 29754 ^T^	NG_042319	NR_119393	NG_060997	LC001756	–
*Caryosporaaquatica*	MFLU 11-1083 ^T^	NG_059058	NR_156408	MH057850	–	–
*Caryosporasubmersa*	MFLUCC 18-1283 ^T^	MN913720	–	–	–	–
*Clematidisitalica*	MFLUCC 15-0084 ^T^	KU842381	KU842380	KU842382	–	–
*Coelodictyosporiumrosarum*	MFLUCC 17-0776 ^T^	MG828991	MG828875	MG829102	MG829195	–
*Corylicolaitalica*	MFLU 19-0500 ^T^	MT554926	MT554925	MT554923	–	MT590776
*Corynesporacassiicola*	CBS 100822	GU301808	–	GU296144	GU349052	GU371742
*Corynesporatorulosa*	CPC 15989 ^T^	KF777207	NR_145181	–	–	–
*Crassimassarinamacrospora*	MAFF 239606 ^T^	LC194344	LC194478	LC194302	LC194389	LC194426
*Crassimassarinamacrospora*	HHUF 30512	LC194343	LC194477	LC194301	LC194388	LC194425
*Crassiperidiumoctosporum*	MAFF 242971 ^T^	LC373108	LC373096	LC373084	LC373120	LC373132
*Crassiperidiumoctosporum*	MAFF 246401	LC373111	LC373099	LC373087	LC373123	LC373135
*Cryptocoryneumjaponicum*	HHUF 30482 ^T^	NG_059035	NR_153938	NG_065118	LC096144	LC194438
*Cryptocoryneumpseudorilstonei*	CBS 113641 ^T^	NG_059036	NR_153941	LC194322	LC096152	LC194446
*Cucurbitariaberberidis*	MFLUCC 11-0387	KC506796	–	KC506800	–	–
*Curvulariaaustriaca*	CBS 102694 ^T^	–	MN688802	–	MN688856	–
*Curvulariaeleusinicola*	USJCC-0005 ^T^	–	MT262877	–	MT432925	–
*Cylindroaseptosporaleucaenicola*	MFLUCC 17-2424 ^T^	MK347966	NR_163333	MK347856	–	–
*Cyclothyriellarubronotata*	CBS 141486 ^T^	KX650544	NR_147651	NG_061252	KX650519	KX650574
*Dacampiahookeri*	GZU 73897	KT383792	–	–	–	–
*Dacampiahookeri*	GZU 81840	KT383795	–	–	–	–
*Dacampiahookeri*	GZU 75980	KT383794	–	–	–	–
*Delitschianypae*	MFLUCC 17-2588 ^T^	–	–	–	MK360049	MK434878
*Dendryphionfluminicola*	MFLUCC 17-1689 ^T^	MG208141	NR_157490	–	MG207992	–
*Deniquelatacassiae*	CMD012A ^T^	OR500088	OR500092	OR500090	OR501827	–
*Dictyocheirosporabannica*	KH 332 ^T^	AB807513	LC014543	AB797223	AB808489	–
*Didymellaexigua*	CBS 183.55 ^T^	MH868977	MH857436	GU296147	–	–
*Didymellarumicicola*	CBS 683.79 ^T^	MH873007	KT389503	–	–	KT389622
*Dothidotthiarobiniae*	MFLUCC 16-1175 ^T^	MK751817	MK751727	MK751762	MK908017	MK920237
*Epicoccumduchesneae*	CGMCC 3.18345 ^T^	KY742249	KY742095	–	–	MT018115
*Epicoccumduchesneae*	CBS 218.81	MN973322	MN972935	–	–	MN983572
*Falciformisporaaquatica*	MFLUCC 18-0212 ^T^	MK063643	MK064216	–	MK099811	–
*Falciformisporatompkinsii*	CBS 200.79 ^T^	MH872968	MH861199	KF015639	KF015685	KF015719
*Fenestellacrataegi*	CBS 144857 ^T^	–	NR_165534	–	MK357555	MK357512
*Fissuromacalami*	MFLUCC 13-0836 ^T^	MF588993	–	NG_062430	MF588975	–
*Flammeascomalignicola*	MFLUCC 10-0128 ^T^	KT324583	KT324582	KT324584	KT324585	KT324586
*Flavomycesfulophazii*	CBS 135761 ^T^	NG_058131	NR_137960	NG_061191	–	–
*Foliophomafallens*	CBS 161.78	GU238074	KY940772	GU238215	–	KC584502
*Fuscostagonosporacytisi*	MFLUCC 16-0622 ^T^	KY770978	–	KY770977	KY770979	–
*Fuscostagonosporasasae*	HHUF 29106 ^T^	AB807548	AB809636	AB797258	AB808524	–
*Fusculinaeucalypti*	CBS 120083 ^T^	DQ923531	DQ923531	–	–	–
*Fusculinaeucalyptorum*	CBS 145083 ^T^	MK047499	NR_161140	–	–	–
*Fusiformisporaclematidis*	MFLUCC 17-2077 ^T^	MT214542	MT310589	MT226661	MT394725	MT394677
*Gordonomycesmucovaginatus*	CMW 22212 ^T^	JN712552	JN712486	–	–	–
*Halojulellaavicenniae*	BCC 20173 ^T^	GU371822	–	GU371830	GU371815	GU371786
*Helminthosporiellastilbacea*	MFLUCC 15-0813 ^T^	MT928157	MT928159	MT928161	MT928151	–
*Hermatomycesamphisporus*	CBS 146610 ^T^	LR812664	LR812664	–	–	–
*Hermatomycesamphisporus*	CBS 146613	LR812662	LR812662	–	LR812657	LR812668
*Hermatomycesamphisporus*	CBS 146614	LR812666	LR812666	–	LR812660	LR812671
*Hermatomycesanomianthi*	MFLUCC 21-0202 ^T^	OK655817	OL413437	–	OM117546	–
*Hermatomycesbifurcatus*	CCF 5900 ^T^	LS398263	LS398263	–	LS398417	LS398344
*Hermatomycesbifurcatus*	CCF 5899	LS398262	LS398262	–	LS398416	LS398343
*Hermatomycesclematidis*	MFLUCC 17-2085 ^T^	MT214556	MT310603	MT226673	MT394735	MT394684
*Hermatomycesconstrictus*	CCF 5904 ^T^	LS398264	LS398264	–	LS398418	LS398345
*Hermatomyceshainanensis*	GZCC 23-0592 ^T^	OR091329	OR098708	–	–	–
*Hermatomycesindicus* (=*H.thailandicus*)	MFLUCC 14-1143 ^T^	KU764692	KU144920	KU712468	–	KU712488
*Hermatomycesindicus* (=*H.thailandicus*)	MFLUCC 14-1144	KU764693	KU144921	KU712469	–	KU712489
*Hermatomycesindicus* (=*H.thailandicus*)	MFLUCC 14-1145	KU764694	KU144922	KU712470	KU872756	KU712490
*Hermatomycesiriomotensis*	KH 361	LC194367	LC194483	–	LC194394	LC194449
*Hermatomycesjinghaensis*	HKAS 112167 ^T^	MW989519	MW989495	–	MZ042642	–
*Hermatomyceskrabiensis* (=*H.chiangmaiensis*)	MFLUCC 16-0249 ^T^	KX525742	KX525750	–	–	KX525754
*Hermatomyceskrabiensis* (=*H.chiangmaiensis*)	MFLUCC 16-2819	KY559394	–	–	–	–
*Hermatomycesmaharashtraensis*	NFCCI 4879 ^T^	NG_241939	NR_189384	–	MZ130659	MZ130660
*Hermatomycesmegasporus*	CCF 5898 ^T^	LS398266	LS398266	–	LS398420	–
*Hermatomycesmegasporus*	CCF 5897	–	LS398265	–	LS398419	LS398346
*Hermatomycesnabanheensis*	KUMCC 16-0149 ^T^	KY766059	KY766058	KY766060	KY766061	–
*Hermatomycespyriformis*	CGMCC 3.27462 ^T^	PP491962	PP491964	–	PP505452	PP505454
*Hermatomycespyriformis*	UESTCC 23.0441	PP491963	PP491965	–	PP505453	PP505455
*Hermatomycesreticulatus* (=*H.subiculosus*)	MFLUCC 15-0843 ^T^	KX259523	KX259521	KX259525	KX259527	KX259529
*Hermatomycesreticulatus* (=*H.subiculosus*)	CCF 5893	LS398267	LS398267	–	LS398421	LS398347
*Hermatomycessphaericoides*	CCF 5908 ^T^	LS398273	LS398273	–	LS398427	LS398352
*Hermatomycessphaericoides*	CCF 5895	LS398270	LS398270	–	LS398424	LS398350
*Hermatomycessphaericus*	PMA 116080	LS398281	LS398281	–	LS398431	LS398356
*Hermatomycessphaericus*	PMA 116081	LS398283	LS398283	–	LS398432	LS398357
*Hermatomycessphaericus*	PRC 4105	–	LS398286	–	–	–
*Hermatomycessphaericus*	PRC 4104	–	LS398278	–	LS398430	LS398355
*Hermatomycessphaericus*	KZP 462	–	LS398287	–	LS398434	LS398359
*Hermatomycessphaericus*	PRM 946201	–	LS398284	–	LS398433	LS398358
*Hermatomycessphaericus*	PRC 4116	–	LS398275	–	–	–
*Hermatomycessphaericus*	PRC 4100	LS398277	LS398277	–	LS398429	LS398354
*Hermatomycessphaericus*	PRC 4106	LS398279	LS398279	–	–	–
*Hermatomycessphaericus*	PMA 116085	–	LS398280	–	–	–
*Hermatomycessphaericus*	PMA 116082	–	LS398285	–	–	–
*Hermatomycessphaericus*	PRC 4117	–	LS398276	–	–	–
*Hermatomycessphaericus*	MFLUCC 17-0373	OL782061	OL782144	OL780526	–	–
*Hermatomycessphaericus*	HKAS 112725	MW989516	MW989492	–	MZ042639	MZ042636
*Hermatomycessphaericus*	HKAS 112166	MW989517	MW989493	–	MZ042640	MZ042637
*Hermatomycessphaericus* (=*H.biconisporus*)	KUMCC 17-0183	MH260296	MH275063	MH260338	MH412771	MH412755
*Hermatomycessphaericus* (=*H.chromolaenae*)	MFLUCC 16-2818	KY559393	–	–	–	–
*Hermatomycessphaericus* (=*H.pandanicola*)	MFLUCC 16-0251	KX525743	KX525751	KX525747	KX525759	KX525755
*Hermatomycessphaericus* (=*H.saikhuensis*)	MFLUCC 16-0266	KX525740	KX525748	–	KX525756	KX525752
*Hermatomycessphaericus* (=*H.saikhuensis*)	MFLUCC 16-0267	KX525741	KX525749	–	KX525757	KX525753
*Hermatomycessphaericus* (=*H.tectonae*)	MFLUCC 14-1140	KU764695	–	NG_063603	KU872757	KU712486
*Hermatomycessphaericus* (=*H.tectonae*)	MFLUCC 14-1141	KU764696	KU144918	KU712466	KU872758	–
*Hermatomycessphaericus* (=*H.tectonae*)	MFLUCC 14-1142	KU764697	KU144919	KU712467	–	KU712487
*Hermatomycestrangensis*	BCC 80741 ^T^	KY790600	KY790598	KY790602	KY790606	KY790604
*Hermatomycestrangensis*	BCC 80742	KY790601	KY790599	KY790603	KY790607	KY790605
*Hermatomycestucumanensis*	CCF 5912	LS398288	LS398288	–	LS398435	LS398360
*Hermatomycestucumanensis*	CCF 5915	LS398290	LS398290	–	LS398437	LS398362
*Hermatomycestucumanensis*	CCF 5913	LS398289	LS398289	–	LS398436	LS398361
*Hermatomycesturbinatus*	MFLUCC 21 0038 ^T^	MW989518	MW989494	–	MZ042641	MZ042638
*Hermatomycesverrucosus*	CCF 5903 ^T^	LS398292	LS398292	–	LS398439	LS398364
*Hermatomycesverrucosus*	CCF 5892	LS398291	LS398291	–	LS398438	LS398363
*Hongkongmycesaquaticus*	MFLUCC 18-1150 ^T^	MN913694	–	MT864302	MT954379	–
*Hypsostromacaimitalense*	GKM1165 ^T^	GU385180	–	–	–	–
*Hypsostromathailandicum*	MFLUCC 21-0057 ^T^	MZ435867	MZ435865	–	–	–
*Jeremyomyceslabinae*	CBS 144617 ^T^	MK442529	MK442589	–	MK442695	MK442665
*Juncaceicolaalpina*	CBS 456.84 ^T^	MH873460	MH861761	KY090699	KF253139	KF252188
*Keissleriellacamporesiana*	MFLUCC 15-0029 ^T^	MN401741	MN401745	MN401743	MN397907	–
*Latoruacaligans*	CBS 576.65 ^T^	NG_058180	–	–	–	–
*Latoruagrootfonteinensis*	CBS 369.72 ^T^	NG_058181	–	–	–	–
*Lentimurisporaurniformis*	MFLUCC 18-0497	MH179144	–	MH179160	MH188055	–
*Lentitheciumclioninum*	HHUF 28199 ^T^	NG_059391	NR_154137	NG_064845	AB808515	–
*Lentitheciumpseudoclioninum*	HHUF 29055 ^T^	NG_059392	AB809633	NG_064847	AB808521	–
*Leptosphaeriachatkalica*	YGS22 ^T^	MW886099	MW886101	MW886100	MW915583	–
*Leptosphaerioidesguizhouensis*	GZAAS 19-4017 ^T^	OP099529	OR225067	OR134435	–	–
*Leptosphaerioidesguizhouensis*	GZAAS 19-4018	OP099530	OR225068	OR134436	–	–
*Leucaenicolaphraeana*	MFLUCC 18-0472 ^T^	MK348003	MK347785	NG_065784	MK360060	MK434867
*Leucaenicolaaseptata*	MFLUCC 17-2423 ^T^	NG_066309	NR_163332	NG_065776	MK360059	MK434891
*Libertasomycesmyopori*	CPC 27354 ^T^	NG_058241	KX228281	–	–	–
*Ligninsphaeriajonesii*	MFLUCC 15-0641 ^T^	KU221037	–	–	–	–
*Ligninsphaeriajonesii*	GZCC 15-0080	KU221038	–	–	–	–
*Lindgomycescigarospora*	G619 ^T^	KX655804	KX655794	KX655805	–	–
*Lindgomycesingoldianus*	ATCC 200398 ^T^	AB521736	NR_119938	NG_016531	–	–
*Longicorpusstriataspora*	MFLUCC 18-0267 ^T^	MK035988	MK035965	MK035973	MK034428	MK034436
*Longiostiolumtectonae*	MFLUCC 12-0562 ^T^	KU764700	KU712447	KU712459	–	–
*Longipedicellataaptrootii*	MFLU 10-0297 ^T^	KU238894	KU238893	KU238895	KU238892	KU238891
*Longipedicellatamegafusiformis*	SJ-KR4 ^T^	MZ538546	MZ538512	–	MZ567090	–
*Lonicericolaqujingensis*	GMBCC1178 ^T^	OM855602	OM855593	OM855616	OM857556	–
*Lophiostomacarpini*	CBS 147279 ^T^	MW750386	NR_173000	–	MW752405	MW752384
*Lophiostomaclavatum*	MFLUCC 18-1316 ^T^	MN274566	–	MN304835	MN328901	–
*Lophiotremaeburnoides*	MAFF 242970 ^T^	LC001707	LC001709	LC001706	LC194403	LC194458
*Magnibotryascomakunmingense*	HKAS 111919 ^T^	MW424785	MW424770	MW424800	MW430106	MW430113
*Magnibotryascomarubriostiolatum*	CBS 140734 ^T^	–	KU601590	–	KU601609	KU601599
*Magnicamarosporiumiriomotense*	HHUF 30125 ^T^	AB807509	AB809640	AB797219	AB808485	–
*Massariainquinans*	CBS 125591 ^T^	MH875187	MH863726	HQ599442	HQ599340	–
*Massarinapandanicola*	MFLUCC 17-0596 ^T^	MG646947	MG646958	MG646979	MG646986	–
*Massarioramusculicolachiangraiensis*	MFLUCC 17-2240 ^T^	MH040228	MH040227	MH040229	–	–
*Massariosphaeriaclematidis*	MFLU 16-0174 ^T^	MT214544	MT310591	MT226663	–	–
*Matsushimamycesbohaniensis*	CBS 140592 ^T^	KR350633	KP765516	–	–	–
*Misturatosphaeriaaurantonotata*	GKM 1238 ^T^	NG_059927	–	–	GU327761	–
*Montagnulaacaciae*	MFLUCC 18-1636 ^T^	ON117298	ON117280	ON117267	ON158093	–
*Montagnulaaquatica*	MFLU 22-0171 ^T^	OP605986	OP605992	OP600504	–	–
*Montagnulaaquatica*	KUNCC 23-14425	OR583116	OR583097	OR583135	OR588088	OR588107
*Morosphaeriamuthupetensis*	NFCC I4219 ^T^	MF614796	MF614795	MF614797	MF614798	–
*Multiloculariabambusae*	MFLUCC 11-0180 ^T^	KU693438	KU693446	KU693442	–	–
*Multiseptosporathailandica*	MFLUCC 11-0183 ^T^	KP744490	KP744447	KP753955	KU705657	–
*Murilentitheciumlonicerae*	MFLUCC 18-0675 ^T^	MK214373	MK214370	MK214376	MK214379	–
*Murisporagalii*	MFLUCC 13-0819 ^T^	KT709175	KT736081	KT709182	KT709189	–
*Muritestudinachiangraiensis*	MFLUCC 17-2551 ^T^	MG602248	MG602247	MG602249	MG602251	MG602250
*Neoastrosphaeriellaphoenicis*	MFLUCC 18-1477 ^T^	MN712339	MN735995	MN699324	MN744232	MN744233
*Neobambusicolamagnoliae*	HKAS 107122 ^T^	ON870389	ON878076	ON870914	–	–
*Neocamarosporiumgoegapense*	CPC 23676 ^T^	KJ869220	KJ869163	–	–	–
*Neocamarosporiumhalophilum*	RSM70 ^T^	–	OR242722	–	OR289930	–
*Neocamarosporiumhalophilum*	BBB RMS57	OR297952	OR297950	–	OR339881	–
*Neohelicascusaquaticus*	KUMCC 19-0107 ^T^	MT627662	MT627719	MT864314	MT954384	–
*Neolophiostomapigmentatum*	MFLUCC 10-0129 ^T^	KT324588	KT324587	KT324589	KT324590	–
*Neomassariafabacearum*	MFLUCC 16-1875 ^T^	KX524145	–	NG_061245	KX524149	–
*Neomassariaformosana*	NTUCC 17-007 ^T^	MH714756	–	MH714759	MH714762	MH714765
*Neomassariahongheensis*	KUMCC 21-0344 ^T^	OL423113	OL477614	OL423115	OL754594	OL754595
*Neomassarinachromolaenae*	MFLUCC 17-1480 ^T^	MT214466	MT214372	MT214419	MT235785	MT235822
*Neomassarinapandanicola*	MFLUCC 16-0270 ^T^	MG298946	MG298945	MG298947	–	–
*Neomassarinathailandica*	MFLU 11-0144 ^T^	NG_059718	NR_154244	–	–	–
*Neomassarinathailandica*	MFLUCC 17-1432	MT214467	MT214373	MT214420	–	–
*Neooccultibambusathailandensis*	MFLUCC 16-0274 ^T^	MH260308	MH275074	MH260348	MH412780	MH412758
*Neophaeosphaeriaphragmiticola*	KUMCC 16-0216 ^T^	MG837009	–	NG_065735	MG838020	–
*Neophaeosphaerialivistonae*	NCYUCC 19-0393 ^T^	OQ437387	OQ437390	OQ437393	–	–
*Neoplatysporoidesaloes*	CPC 36068 ^T^	MN567619	NR_166316	–	–	–
*Neopyrenochaetaannellidica*	MFLU 11-1105 ^T^	MT183502	MT185538	–	–	–
*Neopyrenochaetacercidis*	MFLUCC 18-2089 ^T^	MK347932	MK347718	MK347823	–	MK434908
*Neopyrenochaetachiangraiensis*	MFLUCC 13-0881 ^T^	MT183503	MT185539	MT214975	MT454041	–
*Neopyrenochaetopsishominis*	UTHSC: DI16-238 ^T^	LN907381	LT592923	–	–	LT593061
*Neoroussoellachiangmaiensis*	MFLU 22-0205 ^T^	OQ065735	OQ065738	OQ065736	OQ186448	OQ186450
*Neothyrostromaencephalarti*	CPC 35999 ^T^	MN567613	MN562105	–	MN556831	–
*Neotorulaaquatica*	MFLUCC 15-0342 ^T^	KU500576	KU500569	KU500583	–	–
*Neotorulasubmersa*	KUMCC 15-0280 ^T^	KX789217	KX789214	–	–	–
*Nigrogranaitalica*	MFLU 23-0139 ^T^	OR538591	OR538590	–	OR531366	OR531365
*Occultibambusabambusae*	MFLUCC 13-0855 ^T^	KU863112	KU940123	–	KU940193	KU940170
*Occultibambusajonesii*	GZCC 16-0117 ^T^	KY628322	–	KY628324	KY814756	KY814758
*Ochraceocephalafoeniculi*	CBS 145654 ^T^	MN516774	MN516753	MN516743	MN520149	MN520145
*Ohleriamodesta*	CBS 141480	–	KX650563	KX650513	KX650534	KX650583
*Ohleriamodesta*	WU 36870	–	KX650562	–	KX650533	KX650582
*Omaniahydei*	SQUCC 13750 ^T^	MW077155	MW077146	MW077162	MW075772	MW276077
*Paraconiothyriumkelleni*	CBS 149290 ^T^	NG_229027	NR_185757	OP348926	OP328919	–
*Paradictyoarthriniumaquatica*	MFLUCC 16-1116 ^T^	NG_064501	NR_158861	–	–	–
*Paradictyoarthriniumdiffractum*	MFLUCC 13-0466	KP744498	KP744455	KP753960	–	KX437764
*Paradictyocheirosporatectonae*	AMH 10301 ^T^	MW854647	MW854646	–	MW854832	–
*Paralophiostomahysterioides*	PUFNI 17617	MT912850	MN582758	MN582762	–	MT926117
*Paraleptosphaeriapolylepidis*	MA 57843 ^T^	–	NR_119469	–	–	–
*Paraleptosphaeriapolylepidis*	APA-2999	MK795717	MK795714	MK795720	MK831009	–
*Paramonodictysglobosa*	HKAS 129169 ^T^	OR091331	OR139016	–	OR494045	OR494048
*Parapyrenochaetaprotearum*	CBS 131315	JQ044453	JQ044434	–	–	LT717683
*Periconiadelonicis*	MFLUCC 17-2584 ^T^	NG_068611	–	NG_065770	–	MK434901
*Periconiapseudodigitata*	KT 1395 ^T^	AB807564	LC014591	AB797274	–	–
*Phaeomycocentrosporaxinjangensis*	CGMCC 3.20479 ^T^	OK256190	OK256193	–	–	–
*Phaeoseptummali*	MFLUCC 17-2108 ^T^	MK625197	MK659580	–	MK647990	MK647991
*Phaeoseptumterricola*	MFLUCC 10-0102 ^T^	MH105779	MH105778	MH105780	MH105781	MH105782
*Phaeosphaeriaoryzae*	CBS 110110 ^T^	KF251689	KF251186	GQ387530	–	KF252193
*Pleomonodictyscapensis*	CBS 968.97 ^T^	KY853521	MH862684	–	–	–
*Pleomonodictyscapensis*	DLUCC 1323	MZ420757	MZ420742	–	–	MZ442696
*Pleomonodictysdescalsii*	FMR 12716 ^T^	KY853522	KY853461	–	–	–
*Plenodomuschangchunensis*	CCMJ 5011 ^T^	OL897174	OL996123	OL984031	–	–
*Plenodomuschangchunensis*	CCMJ 5012	OL966928	OL996124	OL984032	–	OL944508
*Pleopunctumclematidis*	MFLUCC 17-2091 ^T^	MT214573	MT310618	–	MT394632	MT394693
*Pleopunctumthailandicum*	MFLUCC 21-0039 ^T^	MZ198896	MZ198894	–	MZ172461	–
*Polyschemasclerotigenum*	UTHSC DI14-305 ^T^	KP769976	KP769975	–	–	–
*Prosthemiumalni*	MFLUCC 17-0240 ^T^	KY815013	KY797636	–	KY815019	–
*Prosthemiumintermedium*	HHUF 30063 ^T^	AB553778	AB554108	–	–	–
*Pseudoasteromassariaaquatica*	MFLUCC 18-1397 ^T^	MN913721	MT627674	MT864322	MT954378	–
*Pseudoastrosphaeriellalongicolla*	MFLUCC 11-0171 ^T^	KT955476	–	–	KT955438	KT955420
*Pseudoastrosphaeriellathailandensis*	MFLUCC 11-0144 ^T^	KT955478	–	KT955457	KT955440	KT955416
*Pseudoberkleasmiumacaciae*	MFLUCC 17-2590 ^T^	NG_066316	NR_163343	NG_065782	MK360073	–
*Pseudoberkleasmiumchiangmaiense*	MFLUCC 17-1809 ^T^	MK131260	MK131259	–	MK131261	–
*Pseudoberkleasmiumchiangmaiense*	MFLU 21-0290	OM065940	OM066271	OM065948	OM102996	OM102997
*Pseudoberkleasmiumchiangmaiense*	MFLUCC 17-2088	MT214585	MT310630	MT226698	MT394643	MT394699
*Pseudoberkleasmiumchiangmaiense*	DLUCC 1655	MZ420759	MZ420744	MZ420749	MZ442693	–
*Pseudoberkleasmiumchiangraiense*	MFLUCC 21-0154 ^T^	OL584200	OL584189	OL606408	OL912943	OL697401
*Pseudoberkleasmiumchiangraiense*	MFLUCC 21-0161	OL584201	OL584190	OL606409	OL912944	OL697402
*Pseudoberkleasmiumchiangraiense*	MFLUCC 21-0162	OL584205	OL584191	OL606410	OL912945	OL697403
*Pseudoberkleasmiumpandanicola*	KUMCC 17-0178 ^T^	MH260304	MH275071	MH260344	–	–
*Pseudochaetosphaeronemachiangraiense*	MFLU 21-0083 ^T^	MZ457922	MZ457923	–	MZ476770	–
*Pseudochaetosphaeronemachiangraiense*	UESTCC 23.0065	OR253260	OR253108	–	OR251160	–
*Pseudochaetosphaeronemairregulare*	CGMCC 3.22458 ^T^	OQ758163	OQ798972	OQ758194	OQ809057	OQ809023
*Pseudochaetosphaeronemairregulare*	CGMCC 3.22461	OQ758162	OQ798971	OQ758193	OQ809056	OQ809022
*Pseudocoleodictyosporasukhothaiensis*	MFLUCC 12-0554 ^T^	KU764710	KU712440	NG_062416	–	KU712493
*Pseudocoleodictyosporathailandica*	MFLUCC 12-0565 ^T^	KU764701	NR_154337	NG_062417	–	KU712494
*Pseudocoleophomaheteropanacicola*	ZHKUCC 23-0880 ^T^	OR365486	OR365456	–	OR700204	–
*Pseudolophiotremaelymicola*	HHUF 28984 ^T^	LC194381	LC194505	LC194339	LC194418	LC194473
*Pseudomassarinaclematidis*	MFLU 16-0493 ^T^	MT214586	MT415397	MT226699	MT394644	MT394700
*Pseudopyrenochaetalycopersici*	FMR 15746 ^T^	EU754205	NR_103581	NG_062728	–	LT717680
*Pseudopyrenochaetaterretris*	FMR 15327 ^T^	LT623216	LT623228	–	–	LT623287
*Pseudotetraploarajmachiensis*	NFCCI 4618 ^T^	MN937204	MN937222	–	–	–
*Pseudoxylomyceselegans*	KT 2887	AB807598	LC014593	AB797308	AB808576	–
*Profundisphaeriafusiformispora*	GZAAS 20-4010 ^T^	–	–	OR134442	OR140432	OR146942
*Profundisphaeriafusiformispora*	GZAAS 20-4012	OR209667	–	OR134443	OR140433	–
*Pyrenochaetopsisleptospora*	CBS 101635 ^T^	GQ387627	JF740262	NG_063097	MF795881	LT623282
*Pyrenochaetopsistabarestanensis*	IBRC:M 30051 ^T^	KF803343	NR_155636	NG_065034	–	–
*Quadricrurabicornis*	HHUF 30023 ^T^	AB524613	AB524797	AB524472	AB524828	–
*Quercicolafusiformis*	MFLUCC 18-0479 ^T^	MK348009	MK347790	MK347898	MK360085	MK434864
*Quercicolaguttulospora*	MFLUCC 18-0481 ^T^	MK348010	MK347791	MK347899	MK360086	–
*Quixadomycescearensis*	HUEFS 238438 ^T^	MG970695	NR_160606	–	–	–
*Roussoellabambusarum*	GMBC 0316 ^T^	ON479892	ON479891	–	ON505017	ON505012
*Roussoellaguttulata*	MFLUCC 20-0102 ^T^	MT734818	MT734821	–	MW022188	MW022187
*Rubroshiraiabambusae*	HKAS 102255 ^T^	MK804658	MK804678	MK804704	MK819218	–
*Rubroshiraiabambusae*	HKAS 102256	MK804659	MK804679	MK804705	MK819219	–
*Salsugineaphoenicis*	MFLU 19-0015 ^T^	MK405280	–	–	MK404650	–
*Salsuginearamicola*	KT 2597.2	GU479801	–	GU479768	GU479862	GU479834
*Salsuginearhizophorae*	MFLU 18-0540 ^T^	MN017851	–	MN017917	–	–
*Seltsamiaulmi*	CBS 143002 ^T^	MF795794	MF795794	MF795794	MF795882	MF795836
*Septoriellachlamydospora*	MFLUCC 15-0177 ^T^	KU163654	KU163658	KU163655	–	–
*Septoriellahibernica*	CBS 145371 ^T^	MK540036	MK539966	–	–	MK540097
*Setoarthopyreniachromolaenae*	MFLUCC 17-1444 ^T^	MT214438	MT214344	MT214392	MT235768	MT235805
*Shiraiabambusicola*	GZAAS 2.0708 ^T^	KC460982	GQ845414	–	–	–
*Shiraiabambusicola*	HKAS 102267	MK804657	MK804677	MK804703	MK819217	MK819237
*Sporormurisporaatraphaxidis*	MFLUCC 17-0742 ^T^	MG829083	MG828971	MG829183	–	–
*Sporormurisporapaulsenii*	MFLUCC 17-1957 ^T^	MK966143	–	MK963075	–	MN023029
*Stagonosporaforlicesenensis*	MFLUCC 15-0054 ^T^	KX655547	KX655557	KX655552	KX655562	–
*Stagonosporaimperaticola*	MFLUCC 15-0026	KY706133	KY706143	KY706138	KY706146	KY706149
*Stemphyliumclematidis*	MFLUCC 14-0937 ^T^	MT214583	MT310628	MT226696	–	–
*Stemphyliumcarpobroti*	CPC 38637 ^T^	MW175395	MW175355	–	–	–
*Striatiguttulanypae*	MFLUCC 18-0265 ^T^	MK035992	MK035969	MK035977	MK034432	MK034440
*Striatiguttulaphoenicis*	MFLUCC 18-0266 ^T^	MK035995	MK035972	MK035980	MK034435	MK034442
*Subglobosporiumtectonae*	MFLUCC 12-0393 ^T^	KU764703	KU712445	KU712464	–	KU712485
*Subglobosporiumtectonae*	MFLUCC 12-0390	KU764702	KU712446	KU712463	–	KU712495
*Sublophiostomathailandica*	MFLUCC 11-0207 ^T^	KX534212	MW136257	KX534218	KX550077	MW088714
*Sublophiostomathailandica*	MFLUCC 11-0185	KX534216	MW136275	KX534222	KX550080	MW088718
*Submersisporavariabilis*	MFLUCC 17-2360 ^T^	MN913682	MT627683	MT864310	–	–
*Submersisporavariabilis*	N-KR15	MZ538561	MZ538527	MZ538575	MZ567103	MZ567114
*Sulcatisporaacerina*	HHUF 30449 ^T^	LC014610	LC014597	LC014605	LC014615	–
*Sulcatisporaberchemiae*	HHUF 29097 ^T^	AB807534	AB809635	AB797244	AB808509	–
*Sulcosporiumthailandica*	MFLUCC 12-0004	KT426563	MG520958	KT426564	–	–
*Teichosporatrabicola*	C134 ^T^	KU601591	KU601591	–	KU601601	KU601600
*Tetraploaaquatica*	MFLU 19-0995 ^T^	MT530452	MT530448	–	–	–
*Tetraploaaquatica*	MFLU 19-0996	MT530453	MT530449	MT530454	–	–
*Thyridariaacaciae*	CBS 138873 ^T^	NG_058127	KP004469	–	–	–
*Thyridariabroussonetiae*	CBS 141481 ^T^	KX650568	KX650568	KX650515	KX650539	KX650586
*Thyrostromajaczewskii*	MFLUCC 18-0787 ^T^	MK765857	MK765856	MK765858	–	–
*Torulacamporesii*	KUMCC 19-0112 ^T^	MN507402	MN507400	MN507401	MN507403	MN507404
*Torulapluriseptata*	MFLUCC 14-0437 ^T^	KY197855	MN061338	KY197862	KY197875	KY197869
*Trematosphaeriagrisea*	CBS 332.50 ^T^	NG_057979	NR_132039	NG_062930	KF015698	KF015720
*Trematosphaeriapertusa*	CBS 122368 ^T^	NG_057809	NR_132040	FJ201991	KF015701	FJ795476
*Tubeufiaabundata* (Tubeufiales)	MFLUCC 17-2024 ^T^	MH558894	MH558769	–	MH550961	MH551095
*Tubeufiaaquatica* (Tubeufiales)	MFLUCC 16-1249 ^T^	KY320539	KY320522	–	KY320556	MH551142
*Tubeufiahainanensis* (Tubeufiales)	GZCC 22-2015 ^T^	OR030835	OR030842	–	OR046679	OR046685
*Tubeufiahainanensis* (Tubeufiales)	GZCC 23-0589	OR066421	OR066414	–	OR058860	OR058853
*Tzeananiataiwanensis*	NTUCC 17-005 ^T^	MH461120	MH461123	MH461126	MH461130	MH461128
*Tzeananiataiwanensis*	NTUCC 17-006	MH461121	MH461124	MH461127	MH461131	MH461129
*Wicklowiaaquatica*	CBS 125634 ^T^	MH875044	OM322822	GU266232	–	GU371813
*Wicklowiafusiformispora*	N-KR1 ^T^	MZ538567	MZ538533	MZ538576	MZ567108	–
*Xenomassariosphaeriaclematidis*	MFLUCC 14-0923 ^T^	MT214571	MT310616	–	MT394630	–
*Xenomassariosphaeriarosae*	MFLUCC 15-0179 ^T^	MG829092	–	MG829192	–	–
*Xenopyrenochaetopsispratorum*	CBS 445.81 ^T^	GU238136	MH861363	NG_062792	–	KT389671
***Xiuguozhangiabroussonetiae***	**MFLUCC 24-0258 ^T^**	**PQ137419**	**PQ137417**	**PQ137421**	**PQ488461**	**PQ488459**
***Xiuguozhangiabroussonetiae***	**MFLUCC 24-0259**	**PQ137420**	**PQ137418**	**PQ137422**	–	**PQ488460**
*Zopfiarosatii*	CBS 427.62 ^T^	NG_066246	NR_160090	–	–	–

– Data unavailable.

Single gene datasets were aligned using MAFFT version 7 by applying the default setting (https://mafft.cbrc.jp/alignment/server/) (Katoh et al. 2019) and trimmed using trimAl (Capella-Gutiérrez et al. 2009). The trimmed datasets were concatenated using SequenceMatrix (Vaidya et al. 2011). Maximum likelihood (ML) phylogeny was conducted in the IQ-TREE webserver (https://iqtree.cibiv.univie.ac.at) using the default parameters and 1000 ultrafast bootstrap replicates (Nguyen et al. 2015). The nucleotide substitution model for each DNA marker was automatically generated. The Bayesian information criterion (BIC) selection results were as follows: Dataset 1 – invgamma for ITS, LSU, *RPB2* and *TEF1*, and gamma for SSU; Dataset 2 – gamma for ITS and SSU, and invgamma for LSU, *RPB2* and *TEF1*.

Bayesian inference (BI) was carried out in MrBayes on XSEDE (version 3.2.7a) in the online CIPRES Portal (https://www.phylo.org/portal2) (Huelsenbeck et al. 2001; Ronquist and Huelsenbeck 2003; Miller et al. 2010). Markov chain Monte Carlo (MCMC) sampling was applied to obtain posterior probabilities (PP). Four Markov chains were run simultaneously for 50,000,000 and 5,000,000 generations for datasets 1 and 2, respectively, with trees sampled every 100^th^ generation. Burn-in was set to 20% and the remaining 80% were used to compute the PP of the consensus trees. Phylogenetic trees were visualised in FigTree version 1.4.4 (Rambaut and Drummond 2014).

### ﻿Genetic distances

To corroborate the phylogenetic placement and evolutionary relationships of the new taxon, intra- and inter-generic genetic distances were computed in MEGA-X by applying the Kimura 2-parameter substitution model, gamma distribution, and pairwise deletion options (Tamura et al. 2013).

## ﻿Results

### ﻿Phylogenetic analyses

Blast searches of LSU, ITS, SSU, *TEF1* and *RPB2* sequences indicated that the two isolates are highly similar to various genera in Pleosporales, including *Atrocalyx*, *Hermatomyces*, *Lophiotrema* and *Pseudoberkleasmium*.

**Figure 1.**
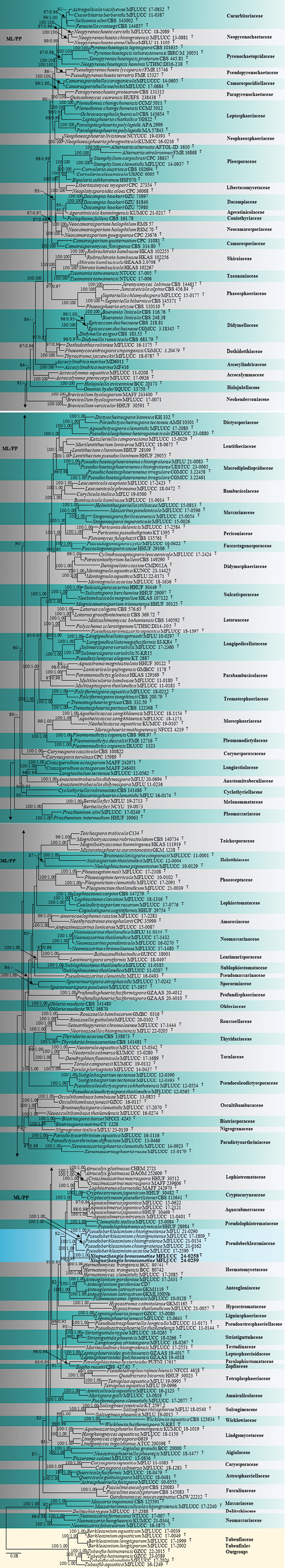
Maximum likelihood analysis (IQ-tree) based on a combined dataset of LSU, ITS, SSU, *TEF1*, and *RPB2* sequences of all families (with representative genera) of Pleosporales. Bootstrap support values (ML ≥ 80%) and Bayesian posterior probabilities (PP ≥ 0.95) are given above the branches or near the nodes as ML/PP. Hyphens (--) indicate bootstrap support values below 80% for ML and posterior probabilities below 0.95. The tree is rooted with *Tubeufiaabundata* (MFLUCC 17-2024), *T.aquatica* (MFLUCC 16-1249), *T.hainanensis* (GZCC 22-2015 and GZCC 23-0589), *Berkleasmiumaquaticum* (MFLUCC 17-0049 and MFLUCC 17-0039) and *B.longisporum* (MFLUCC 17-1999 and MFLUCC 17-2002) (Tubeufiales). Type, ex-type, and reference strains are denoted with ^T^. Our isolates are in bold font. The different colour blocks indicate the families to which the taxa belong.

Dataset 1 consisted of 4250 characters (LSU = 1–846, ITS = 847–1355, SSU = 1356–2359, *TEF1* = 2360–3257, and *RPB2* = 3258–4250), which was analysed to depict relationships at a higher taxonomic level for *Xiuguozhangia* (Fig. 1). Outgroup taxa were selected from Tubeufiales. The log-likelihood of the consensus tree (Fig. 1) was -143487.839. The average standard deviation of split frequencies at the end of the total MCMC generations converged to 0.0092.

**Figure 2.**
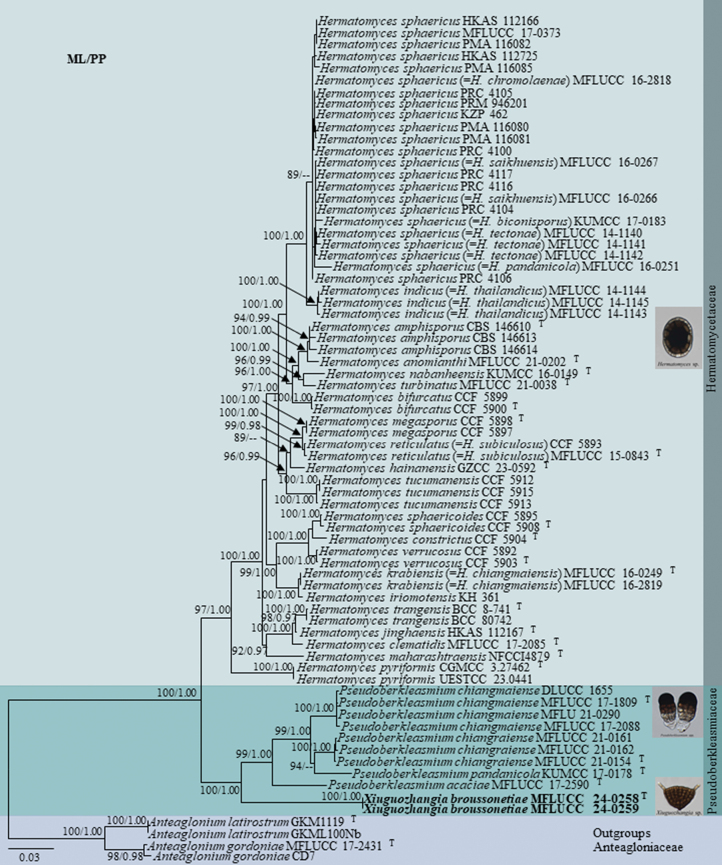
Maximum likelihood analysis (IQ-tree) based on the combined LSU, ITS, SSU, *TEF1* and *RPB2* sequences of *Xiuguozhangia*, *Pseudoberkleasmium* and *Hermatomyces*, generated from dataset 2. Bootstrap support values (ML ≥ 80%) and Bayesian posterior probabilities (PP ≥ 0.95) are given above the branches or near the nodes as ML/PP. Hyphens (--) indicate bootstrap support values below 80% for ML and posterior probabilities below 0.95. The tree is rooted with *Anteagloniumgordoniae* (MFLUCC 17-2431 and CD7) and *A.latirostrum* (GKM1119 and GKML100Nb) (Anteagloniaceae, Pleosporales). Type, ex-type, and reference strains are denoted with ^T^. The new isolates are in bold font. The different colour blocks indicate the families to which the taxa belong.

Dataset 2 comprised 4285 characters (LSU = 1–833, ITS = 834–1344, SSU = 1345–2350, *TEF1* = 2351–3272, and *RPB2* = 3273–4285), and was used to infer the inter-generic relationships of *Xiuguozhangia*, which comprised multiple strains for each genus (Fig. 2). Based on the results from dataset 1, four taxa belonging to Anteagloniaceae (Pleosporales) were selected as outgroups as they are phylogenetically closely related to Pseudoberkleasmiaceae and Hermatomycetaceae. The log-likelihood of the consensus tree (Fig. 2) was -17829.366. The average standard deviation of split frequencies at the end of the total MCMC generations converged to 0.0031.

The two strains of *Xiuguozhangiabroussonetiae* (MFLUCC 24-0258 and MFLUCC 24-0259) grouped with 100% ML and 1.00 PP support. This subclade formed a separate sister lineage to *Pseudoberkleasmium* with 100% ML and 1.00 PP support (Figs 1, 2). Single and combined gene trees from datasets 1 and 2 indicate that our species is phylogenetically most closely related to *Pseudoberkleasmium*, and its placement in Pseudoberkleasmiaceae is supported by maximum bootstrap support and posterior probability in both combined data trees.

### ﻿Genetic distances

Based on the generic relationship depicted in Figs 1, 2, we computed the group mean distances between *Hermatomyces* spp. (group 1), *Pseudoberkleasmium* spp. (group 2) and *Xiuguozhangia* spp. (group 3) across ITS and LSU markers. The difference in the genetic distances across both markers is given in Table 2.

**Table 2.** Group mean genetic distances (%) in *Hermatomyces* spp., *Pseudoberkleasmium* spp., and *Xiuguozhangia* spp. across ITS (511 bp) and LSU (833 bp).

DNA markers	Groups	Group 1: *Hermatomyces* spp.	Group 2: *Pseudoberkleasmium* spp.
**ITS**	**Group 1**: *Hermatomyces* spp.	0	–
	**Group 2**: *Pseudoberkleasmium* spp.	9.052	0
	**Group 3**: *Xiuguozhangia* spp.	10.62834205	13.25008949
**LSU**	**Group 1**: *Hermatomyces* spp.	0	–
	**Group 2**: *Pseudoberkleasmium* spp.	3.1615	0
	**Group 3**: *Xiuguozhangia* spp.	4.1404	3.8669

### ﻿Taxonomy

#### ﻿ Xiuguozhangia broussonetiae

Taxon classificationFungiPleosporalesPseudoberkleasmiaceae

Gomdola, Jayaward. & K.D. Hyde sp. nov.

A6D53491-3A27-5623-88C3-79B9A12646A5

Index Fungorum: IF901943

Facesoffungi Number: FoF16323

Fig. 3

##### Holotype.

MFLU 24-0227.

##### Etymology.

The specific epithet refers to the host genus, *Broussonetia*, from which the species was isolated.

##### Description.

Saprobic on decaying stems of *Broussonetiapapyrifera*. Sexual morph not observed. Asexual morph on substrate. Hyphomycetous. ***Colonies*** on the substrate effuse, hairy, olivaceous to dark brown, appearing velvety due to numerous conidiophores. ***Mycelium*** semi-immersed or immersed, composed of septate, branched, smooth, hyaline or pale brown to brown hyphae. ***Conidiophores*** (430–)550–750(–890) µm long (x– = 681 µm, n = 10), 15–24(–34) µm wide (x– = 21.8 µm, n = 10) at the base, 11–14(–16) µm wide (x– = 12.3 µm, n = 10) in the middle, 7–10 µm(–12) wide (x– = 7.8 µm, n = 10) at the apex, rarely branched, macronematous, mononematous, differentiated, smooth, thick-walled, erect, straight or flexuous, brown to dark brown, wider at the base and ocasionally conical at the apex, comprising 12–17 septa. ***Conidiogenous cells*** 5–12 µm long × 4–9 µm wide (x– = 7.4 × 6.2 µm, n = 10), holoblastic, enteroblastic, integrated, smooth-walled, ovoid to ampulliform, hyaline or brown to dark brown, occurring terminally or intercalary on conidiophores, with up to four successive percurrent proliferations, with new conidiogenous cells developing on subtending cells. ***Conidia*** 25–40 µm long × 30–60 µm wide (x– = 35 × 47 µm, n = 30), width measured between the two extremities of the apices, solitary, dictyospored and cheirospored, fan-shaped to cheiroid, lenticular in edge view, occasionally apically 2–3-lobed, dark brown to olivaceous brown, dictyoseptate, with up to 15 columns of cells radiating from a protuberant basal cell 2–3 µm wide, septa obscured by dark pigmentation, and with 1–3 apical appendages (rarely without appendages). ***Apical appendages*** (2–)6–16(–20) µm long (x– = 10.9 µm, n = 10), 4–5 µm wide (x– = 4.4 µm, n = 10) at the base, 4–5 µm wide (x– = 4.1 µm, n = 10) at the apex, mostly short and untapered, sometimes long and tapering, arising from the sides of the outermost rows of cells of the conidium, pale brown to brown, becoming hyaline and rounded at the tips, and consisting of 1–5 septa.

##### Culture characteristics.

On PDA, colony circular with lobate to crenated margin, reaching 25 mm diam. after 28 days incubated at 25 °C, greyish white to olivaceous brown from center to edge, fluffy, raised to convex, penetrating the media and displaying a dark brown colour around the media.

##### Material examined.

Thailand • Chiang Rai Province, Mae Fah Luang University Botanical Garden, on decaying stems of *Broussonetiapapyrifera* (Moraceae), 19 May 2023, D. Gomdola, F2-A (MFLU 24-0227, ***holotype***), ex-type MFLUCC 24-0258.

##### Additional material examined.

Thailand • Chiang Rai Province, Mae Fah Luang University Botanical Garden, on decaying stems of *Broussonetiapapyrifera* (Moraceae), 19 May 2023, D. Gomdola, F2-B (MFLU 24-0228), living culture MFLUCC 24-0259.

##### GenBank accession numbers.

MFLUCC 24-0258; ITS = PQ137417, LSU = PQ137419, SSU = PQ137421, *RPB2* = PQ488459 and *TEF1* = PQ488461; MFLUCC 24-0259; ITS = PQ137418, LSU = PQ137420, SSU = PQ137422 and *RPB2* = PQ488460.

##### Notes.

Our two isolates (MFLUCC 24-0258 and MFLUCC 24-0259) group together with 100% ML and 1.00 PP support, and this subclade is sister to *Pseudoberkleasmium* species with 100% ML and 1.00 PP support (Figs 1, 2).

**Figure 3.**
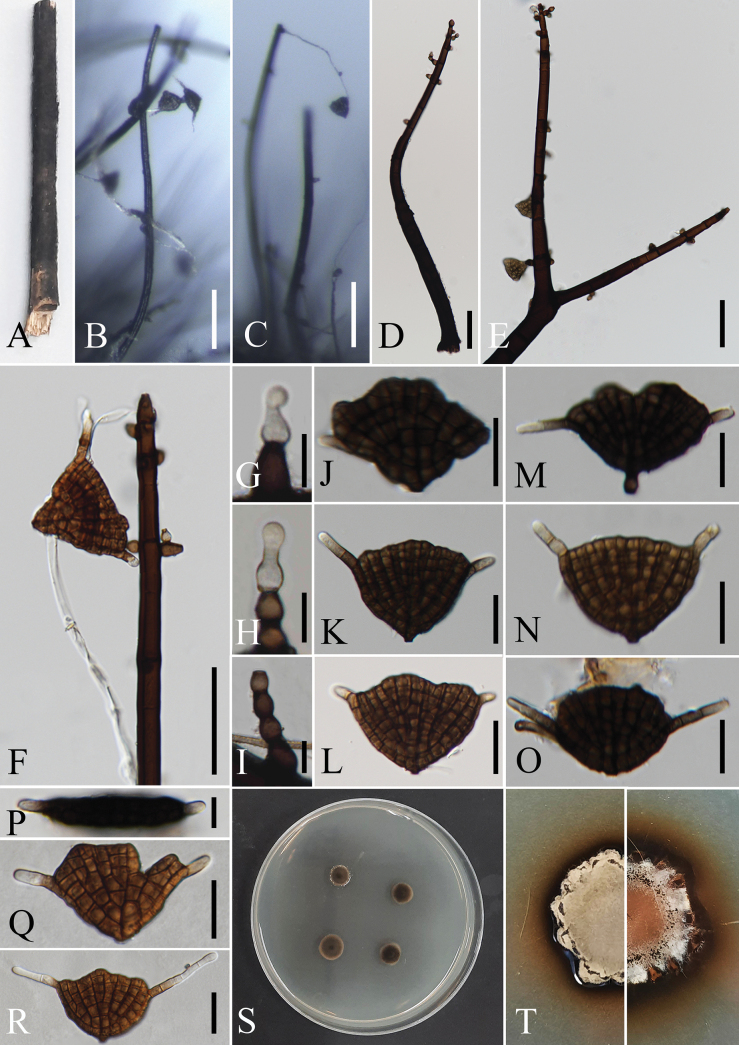
*Xiuguozhangiabroussonetiae* (MFLUCC 24-0258) **A** substrate (*Broussonetiapapyrifera*) **B, C** colonies on the substrate **D–F** conidiophores and attachment of conidia **G–I** conidiogenous cells with percurrent proliferations **J–O, Q, R** conidia bearing appendages **P** top view of a conidium **S** top view of colonies on PDA after 7 days **T** top and reverse colonies on PDA after 28 days. Scale bars: 100 µm (**B, C**); 50 µm (**D–F**); 10 µm (**G–I, P**); 20 µm (**J–O, Q, R**).

A close comparison of the morphological characters across the existing *Xiuguozhangia* species is collated and presented in Table 3 to support the establishment of the new species, *X.broussonetiae*.

**Table 3.** Phenotypic comparison of *Xiuguozhangia* species. Features that depict *X.broussonetiae* from other *Xiuguozhangia* species are in bold.

**Species and characters**		***X.appendiculata***	***X.broussonetiae***	***X.indica***	***X.macrospora***	***X.punicae***	***X.rhaphidophorae***	***X.rosae***
**Colonies on the natural substrates**	**Features**	Effuse, hairy	Effuse, appearing velvety due to numerous conidiophores	Effuse, hairy	Effuse, hairy, growing in association with species of lichen	Effuse, hairy	Effuse, hairy	Effuse, hairy
	**Colour**	Olivaceous to dark brown	Olivaceous to dark brown	Olivaceous to dark brown	Olivaceous to dark brown	Olivaceous to dark brown	Olivaceous to dark brown	Olivaceous to dark brown
**Conidiophores**	**Size (µm)**	Up to 620 µm long, 18–35 µm wide at the base, 13–18 µm wide in the middle, 7–11.5 µm wide at the apex	(430–)550–750**(–890) µm long**, 15–24(–34) µm wide at the base, 11–14(–16) µm wide in the middle, 7–10 µm(–12) wide at the apex	Up to 530 µm long, 13–15 µm wide at the base, 10–12 µm wide in the middle, 3.6–5 µm wide at the apex	160–340(–570) µm long, 12–21 µm wide at the base, 11–14 µm wide in the middle, 6–7.5 µm wide at the apex	Up to 550 µm long, 20–30 µm wide at the base, 10–17 µm wide in the middle, 5–8 µm wide at the apex	Up to 630 µm long, 18–25 µm wide at base, 10–15 µm wide in the middle, 4–7 µm wide at apex	Up to 730 µm long, 12–20 µm wide at the base, 7–14 µm wide in the middle, 5–9 µm wide at the apex
	**Features**	Mononematous, erect, straight or flexuous, unbranched, smooth, thick-walled, comprising 4–8 septa	Macronematous, mononematous, erect, straight or flexuous, **rarely branched**, smooth, thick-walled, wider at the base and ocasionally conical at the apex, comprising **12–17 septa**	Mononematous, basal portion sheath-like, erect, straight, unbranched, smooth, thick-walled, comprising 4–13 septa	Mononematous, erect, straight, unbranched, smooth, thick-walled, comprising 3–8 septa	Macronematous, mononematous, erect, straight or flexuous, unbranched, smooth, thick-walled, comprising 5–11 septa	Macronematous, mononematous, erect, straight or flexuous, unbranched, smooth, thick-walled, comprising 10–15 septa	Macronematous, mononematous, erect, straight or flexuous, unbranched, smooth, thick-walled, comprising 8–14 septa
	**Colour**	Dark brown	Brown to dark brown	Brown, becoming paler towards the apex	Brown	Not mentioned	Not mentioned	Not mentioned
**Conidiogenous cells**	**Size (µm)**	Not mentioned	5–12 µm long, 4–9 µm wide	7.5–13.5 × 4.2–7.4 µm	9–19 × 7.5–10.0 µm	Not mentioned	Not mentioned	Not mentioned
	**Features**	Holoblastic, integrated, terminal, sometimes lateral, truncate after conidium secession	Holoblastic, enteroblastic, integrated, terminal or intercalary, smooth-walled, ovoid to ampulliform, percurrently proliferating; sometimes new condiogenous cells developing on subtending cells	Flask-shaped, integrated, terminal and lateral, truncate after conidial secession, proliferating percurrently up to 4 times	Flask-shaped, integrated, terminal, truncate after conidial secession, proliferating percurrently up to 5 times	Monotretic, integrated, terminal, sometimes lateral, with up to five successive percurrent proliferations	Monotretic, integrated, terminal and lateral	Monotretic, integrated, terminal and lateral, with up to three successive percurrent proliferations
	**Colour**	Not mentioned	**Hyaline** or brown to dark-brown	Brown	Brown	Not mentioned	Not mentioned	Not mentioned
**Conidia**	**Size (µm)**	50–80 µm long, 60–90 µm wide	25–40 µm long, 30–60 µm wide	34–44 µm long, 39–52 µm wide	(73–)81–120(–135) µm long, (51–)67–94(–106) µm wide	50–65 µm long, 58–95 µm wide	27–41 µm long, 30–43 µm wide	45–50 µm long, 53–76 µm wide
	**Features**	Fan-shaped, lenticular in edge view, 2–3-lobed, dictyoseptate, with rows of cells radiating from a protuberant basal cell 6–7.5 µm wide	Dictyospored and cheirospored, fan-shaped to cheiroid, lenticular in edge view, occasionally apically 2–3-lobed, dictyoseptate, with up to 15 columns of cells radiating from a protuberant basal cell **2–3 µm wide**	Fan-shaped, sometimes 2–5-lobed, lenticular in edge view, dictyoseptate, with up to 15 rows of cells, basal cell protuberant, 3.7–5 µm wide	Campanulate or fan-shaped, sometimes apically 2–3-lobed, lenticular in edge view, dictyoseptate, smooth, with up to 18 vertical rows of cells, basal cell protuberant, 7–9 µm wide	Fan-shaped, sometimes 2–3-lobed, lenticular in edge view, dictyoseptate, with up to 18 rows of cells radiating from a protuberant basal cell 4.5–6.5 µm wide	Fan-shaped, sometimes lobed, lenticular in edge view, dictyoseptate, with eight rows of cells radiating from a protuberant basal cell 2.5–5 µm wide	Fan shaped, sometimes 2–3-lobed, lenticular in edge view, dictyoseptate, with 20 rows of cells radiating from a protuberant basal cell 3–5 µm wide
	**Colour**	Dark brown	Dark brown to olivaceous brown	Brown	Brown	Dark brown	Dark brown	Dark brown
**Apical appendage(s)**	**Size (µm)**	75–120 µm long, 4–6 µm wide, tapering to 1.5–2 µm wide	(2–)6–16(–20) µm long, 4–5 µm wide at the base and apex	15–30 µm long, 3–4.4 µm wide at the base, tapering to 2.4–2.8 µm wide	(33–)52–74(–126) µm long, 6.5–8.0 µm wide at the base	24–99 µm long, 3–5.5 µm wide, tapering to 1–1.5 µm wide		15–35 µm long, 3–4 µm wide, tapering to 2–3 µm wide
	**Features**	2–4 appendages, tapering, 3–5-septate	Without or with 1–3 appendages, rounded at the tips, **mostly untapered**, sometimes tapering 1–5-septate	2 appendages (Up to 4), tapering, 1–2-septate	2–3 appendages (rarely up to 4), tapering, 1–3(–6)-septate	2 appendages (rarely up to 3), smooth, tapering, 0–3-septate	Appendages absent	2 appendages, tapering, 1–2-septate
	**Color**	Brown, and apically hyaline	Pale brown to brown, becoming hyaline at the tips	Brown, and apically hyaline	Brown, and apically hyaline	Brown, and apically hyaline	-	Brown, and apically hyaline
**Host(s)**		On dead twigs of an unknown host	On decaying stems of *Broussonetiapapyrifera*	On dried bamboo culms	On dead bamboo stem	On dead branches of *Punicagranatum*	On dead branches of *Rhaphidophoradecursiva*	On dead branches of *Rosachinensis*
**Distribution(s)**		India	Thailand	India	India	China	China	China
**References**		(Bhat and Kendrick 1993)	This study	Sureshkumar et al. (2005)	Dubey and Jash (2024)	Zhang et al. (2009), Zhang et al. (2014)	Zhang et al. (2009, 2014)	Zhang et al. (2009), Zhang et al. (2014)

A summary of the main findings from the morphological assessment is presented below:

1. The conidiophores of our species, *Xiuguozhangiabroussonetiae*, are longer than all other *Xiuguozhangia* taxa (up to 890 μm long), comprising numerous septa (up to 17). Furthermore, conidiophores of other *Xiuguozhangia* species are unbranched, while those of *X.broussonetiae* are sometimes branched (Fig. 3E).
2. The conidia of *Xiuguozhangiabroussonetiae* (Figs 3J–O, Q, R) consist of up to 15 columns of cells radiating from a protuberant basal cell that is smaller (2–3 μm wide) compared to those of other species.
3. The appendages of *X.broussonetiae* are mostly untapered (Figs 3N, 3P–R) and sometimes taper towards the tips (Fig. 3B), whereas all other *Xiuguozhangia* species have only tapering appendages.
4. *Xiuguozhangiabroussonetiae* differs from all extant species but has a close morphological overlap with *X.rosae*. However, the primary feature that demarcates *X.broussonetiae* from *X.rosae* is the number and features of the apical appendages. Up to three appendages were observed in *X.broussonetiae* (sometimes seen without appendage), while *X.rosae* has two appendages. In addition, the appendages of *X.broussonetiae* are 1–5-septate, while those of *X.rosae* display one to two septa.
5. *Xiuguozhangiabroussonetiae* has percurrently proliferating, enteroblastic conidiogenous cells that sometimes produce new conidiogenous cells on subtending cells (Fig. 3D–I). This feature has not been observed in other species of the genus.

Based on these morphological differences, we conclude that our taxon is distinct from the existing *Xiuguozhangia* species.

## ﻿Discussion

The number of hyphomycetes introduced over the past decade has increased substantially, indicating that their diversity is rather high (Hyde et al. 2020, 2023, 2024a, b; Bhunjun et al. 2022; Manawasinghe et al. 2022; Calabon et al. 2023; Liu et al. 2024b; Tian et al. 2024; Zhang et al. 2024). Hyphomycetes have a ubiquitous distribution in aquatic and terrestrial habitats, occurring on different substrates in tropical and subtropical regions (Seifert et al. 2011; Bao et al. 2021; Wu and Diao 2022, 2023; Dong et al. 2023; Senanayake et al. 2023; Yang et al. 2023; Liu et al. 2024a, b). Several hyphomycetes are rather speciose, for example, *Cladosporium*, *Dictyosporium*, *Helminthosporium*, and *Sporidesmium* (Shenoy et al. 2006; Hu et al. 2023; Yang et al. 2023). However, the hyphomycete genus, *Xiuguozhangia*, does not appear to be species-rich, and no studies have reported its occurrence in Thailand. This study introduces a new species, *Xiuguozhangiabroussonetiae*, from *Broussonetiapapyrifera* in northern Thailand.

There are more than 30,000 fungal species that exhibit the asexual morph, belonging to 2265 hyphomycetous genera (Wijayawardene et al. 2022b; Jayawardena et al. 2023; Perera et al. 2023; Ma et al. 2024; Zhang et al. 2024). The *Xiuguozhangia* species reported so far occur only in their asexual morph as saprobic hyphomycetes. A probable explanation for their asexual morph occurrence might be for survival, as conidia can be produced in large amounts and are easily released for dispersal compared to ascospores (Gilbert and Parker 2023). Their sexual morphs have not been reported yet, and no research has been conducted to link their asexual and sexual morphs, possibly due to the lack of molecular data and their availability in cultures. To date, all *Xiuguozhangia* taxa have been documented from Asia (China, India and Thailand – this study). We anticipate discovering more species in this genus, as well as the sexual morph, especially in high biodiversity areas like China and Thailand, as these taxa have not yet been extensively collected, isolated, and studied.

In the phylogenetic analysis of dataset 1, which included representative taxa from all families of Pleosporales, *Xiuguozhangia* forms a sister lineage to *Pseudoberkleasmium*, with maximum statistical support in both methods of analysis (Fig. 1). Pleosporales taxa were accommodated in over 90 families (with and without molecular data), and have a worldwide distribution in terrestrial and aquatic habitats (Hongsanan et al. 2020; Bhunjun et al. 2021; Jiang et al. 2021; Wijayawardene et al. 2020, 2022a; Yang et al. 2022; Tang et al. 2023; Hyde et al. 2024a, b; Pem et al. 2024; Wei et al. 2024). In this study, we maintain 95 families in this order: 92 with DNA sequence data and two without (Diademaceae and Lizoniaceae). Additionally, we exclude Mycoporaceae (*Mycoporum* spp.) from the analysis due to its divergent sequences and long branches in the preliminary phylogenetic trees. Further research is needed to determine its placement within Pleosporales. Arthopyreniaceae was classified under Pleosporales in Wijayawardene et al. (2022a) and Pem et al. (2024), but we do not retain it in this order as its taxa cluster basal to Pleosporales (results not shown), similar to the findings by Thiyagaraja et al. (2021). We also retain Sublophiostomataceae within Pleosporales (Hongsanan et al. 2021), but it was not included in Pem et al. (2024). In our phylogenetic analysis, most families in Pleosporales are monophyletic, thereby further supporting their rank. A few families [e.g., Halotthiaceae (87% ML); Pseudomassarinaceae; and Testudinaceae (81% ML)] lack strong statistical support but have consistently been recovered as distinct groups in previous studies using different datasets and/or methods of analysis (Crous et al. 2018; Yang et al. 2022), suggesting that these are separate lineages distinct from other families. However, available genetic markers and DNA sequence data may not be sufficient to resolve their familial relationships with high confidence.

*Xiuguozhangia* is sister to *Pseudoberkleasmium* and closely related to *Hermatomyces* (Figs 1, 2). Given that we assigned *Xiuguozhangia* in Pseudoberkleasmiaceae based on the analysis of dataset 1, we analysed dataset 2 to further validate its taxonomic placement, which was based on multiple strains representing all species of *Hermatomyces* and *Pseudoberkleasmium*. Since ITS and LSU are important genetic markers to delimit fungal genera, we computed the group mean distances across these gene regions between *Hermatomyces* spp., *Pseudoberkleasmium* spp. and *Xiuguozhangia* spp. Considering the inter-generic nucleotide differences between each group (Table 2), the establishment of *Xiuguozhangia* as a distinct genus from *Pseudoberkleasmium* is supported.

The characterisation of *Xiuguozhangia* as a separate genus from *Pseudoberkleasmium* is further corroborated based on their distinct features. *Xiuguozhangia* differs from *Pseudoberkleasmium* in their colonies on natural substrates, and conidiophore, conidiogenous cell and conidial features (Zhang et al. 2014; Tibpromma et al. 2018). *Xiuguozhangia* species are depicted by dictyoseptate, campanulate, cheiroid, and brown conidia comprising multiple layers of cells, either with or without apical appendages (Zhang et al. 2014). However, *Pseudoberkleasmium* species are characterised by broadly ellipsoidal to obovoid, flattened, one-cell thick, muriform, and brown olivaceous green conidia without any apical appendage (Tibpromma et al. 2018). Conidial images of *Pseudoberkleasmium* and *Hermatomyces* are illustrated in Fig. 2. Since other species of *Xiuguozhangia* do not have molecular data, we were unable to compare the inter-species nucleotide differences, and we had to rely solely on morphology to establish our new species, *X.broussonetiae*.

With respect to the inter-species morphological differences in the genus, the new species, *X.broussonetiae*, differs from all extant species (Table 3), but exhibits overlapping features with *X.rosae*. Nonetheless, *X.broussonetiae* differs from *X.rosae* in terms of the number and characters of the apical appendages, as outlined in the note section above. Other observed differences are given in Table 3. *Xiuguozhangia* was classified as Ascomycota genus *incertae sedis* in the latest outline by Wijayawardene et al. (2022a), as it was difficult to propose its precise familial placement without the availability of DNA sequence data. Since our study is the first to provide molecular data, we refer *Xiuguozhangia* in Pseudoberkleasmiaceae. Hyde et al. (2019) initially introduced this family as monotypic to accommodate *Pseudoberkleasmium* species based on morphology and molecular data. *Pseudoberkleasmium* was described by Tibpromma et al. (2018) based on morphology and multigene phylogenetic analyses. *Pseudoberkleasmium* taxa resemble those of *Berkleasmium*, but *Berkleasmium* belongs to Tubeufiales (Tibpromma et al. 2018; Wijayawardene et al. 2022a; this study). Besides *Pseudoberkleasmium*, we treat *Xiuguozhangia* as a member of Pseudoberkleasmiaceae based on our inferred phylogenies. Upon the addition of *X.broussonetiae* to the genus, there are seven species of *Xiuguozhangia*, sharing the same holoblastic conidial ontogeny as a generic feature.

Due to the limited taxon sampling and analysis, and the lack of comprehensive DNA sequence data, there remains uncertainty in using conidial ontogeny as a basis for distinguishing *Xiuguozhangia* from *Piricaudiopsis*. As such, we refrain from drawing further conclusions regarding their taxonomic separation at this stage. Instead, a more effective approach would involve extensive sampling across diverse localities and habitats, particularly those that support the growth of hyphomycetes. Coupled with a systematic acquisition of DNA sequence data, these efforts would provide a more robust support for classifying *Xiuguozhangia* species and other poorly studied Pleosporalean taxa (Liu et al. 2024b; Pem et al. 2024). Moreover, a thorough re-examination of the type species of each genus is essential to ascertain whether these two genera are indeed distinct or if they might represent a single taxonomic entity.

## Supplementary Material

XML Treatment for Xiuguozhangia broussonetiae

## ﻿Additional information

### Author ORCIDs

Deecksha Gomdola https://orcid.org/0000-0002-0817-1555

Rajesh Jeewon https://orcid.org/0000-0002-8563-957X

Eleni Gentekaki https://orcid.org/0000-0002-3306-6714

Ruvishika S. Jayawardena https://orcid.org/0000-0001-7702-4885

Kevin D. Hyde https://orcid.org/0000-0002-2191-0762

Fatimah Alotibi https://orcid.org/0000-0003-3629-5755

### Data availability

All data generated or analysed during this study are included in this published article.
